# RNA editing differently affects protein-coding genes in D. melanogaster and H. sapiens

**DOI:** 10.1038/srep11550

**Published:** 2015-07-14

**Authors:** Luigi Grassi, Guido Leoni, Anna Tramontano

**Affiliations:** 1 Department of Physics, Sapienza University of Rome, Piazzale Aldo Moro 5, 00185 Rome, Italy; 2 Instituto Pasteur-Fondazione Cenci Bolognetti, Piazzale Aldo Moro 5, Rome 00185, Italy

## Abstract

When an RNA editing event occurs within a coding sequence it can lead to a different encoded amino acid. The biological significance of these events remains an open question: they can modulate protein functionality, increase the complexity of transcriptomes or arise from a loose specificity of the involved enzymes. We analysed the editing events in coding regions that produce or not a change in the encoded amino acid (nonsynonymous and synonymous events, respectively) in D. melanogaster and in H. sapiens and compared them with the appropriate random models. Interestingly, our results show that the phenomenon has rather different characteristics in the two organisms. For example, we confirm the observation that editing events occur more frequently in non-coding than in coding regions, and report that this effect is much more evident in H. sapiens. Additionally, in this latter organism, editing events tend to affect less conserved residues. The less frequently occurring editing events in Drosophila tend to avoid drastic amino acid changes. Interestingly, we find that, in Drosophila, changes from less frequently used codons to more frequently used ones are favoured, while this is not the case in H. sapiens.

RNA editing is a post-transcriptional process that introduces changes in RNA transcripts encoded by genome sequences. It was first observed more than 25 years ago in kinetoplastid protozoa. The insertion or deletion of many uridine nucleotides in the mitochondrial mRNA of these trypanosomes has functional effects at the level of the edited proteins^1^. In higher eukaryotes, the Adenosine Deaminase Acting on RNA (ADAR) enzymes mediate the most prevalent type of RNA editing. These enzymes catalyse the conversion of adenosines to inosines (A to I editing) in double-stranded RNA (dsRNA) substrates^2^. When these modifications occur in protein coding exons they can cause nonsynonymous changes leading to a different encoded residue^3^. Some of these events have been reported in ligand- and voltage-gated ion channels and neurotransmitter receptors in both invertebrates and vertebrates^4^,^5^,^6^,^7^ and they can modulate protein functionality. Furthermore Garret *et al.*^8^ showed that squids from different thermal environments edit their K^+^ channels, thus suggesting an intriguing relationship between RNA editing and physical environment. Many studies associate aberrant RNA editing with a wide range of diseases such as cancer, neurological diseases, viral infections and autoimmune disorders (for detailed reviews see^9^,^10^,^11^ ). The advent of the RNA-Seq technology provided a more detailed description of the eukaryotic transcriptomes and highlighted a frequency of RNA editing higher than previously estimated in various organisms, including fly, human, mouse and squid^12^,^13^,^14^. All these previously unreported events add further complexity to the eukaryotic genomes, but at the same time, make the biological effects of RNA-editing in coding regions less easy to interpret.

Enzymes belonging to the ADAR family are highly conserved in vertebrates and their activity is important for normal development of mammals^15^,^16^,^17^. The enzyme conservation does not mirror the conservation of the edited targets: a recent study compared the mouse and human edited sites and only a small fraction of them were found to be conserved within mammalian evolution^18^. This raises the question of the functional significance of a conserved mechanism that acts on different targets in different organisms.

Here we focus on editing events affecting the coding part of the genome using as model D. melanogaster, an organism for which the editing phenomenon in coding sequences is well documented^13^,^19^,^20^. The comparison of our results with those obtained by performing the same analysis in H. sapiens suggests that the modality and effects of the ADAR-mediated editing in protein coding genes is rather different in the two organisms.

## Results

In order to avoid biases due to particular experimental set-ups or to specific genes or tissues, we selected, for D. melanogaster, data derived from the study of Laurent *et al.* This consists of 3581 editing events identified through single-molecule sequencing (SMS) that have been extensively validated with Sanger sequencing. The included data overlap with previous reported large-scale analyses^12^,^13^,^20^. The human dataset is derived from the Darned database^21^,^22^ filtered as described in Methods.

### Localization of RNA editing in transcripts

Previous studies reported that many editing events occur at the level of coding regions (CDS) in D. melanogaster^13^,^19^ while this is not the case in human^23^,^24^,^25^ where a large fraction of editing events affects the untranslated part of the mature transcripts.

These observations were derived using different strategies to map editing events onto the reference transcriptomes and their statistical significance was not thoroughly assessed.

We calculated the frequency of edited coding and noncoding nucleotides with respect to the total number of coding and noncoding transcribed adenosines for each of the datasets under examination.

**Figure 1:**
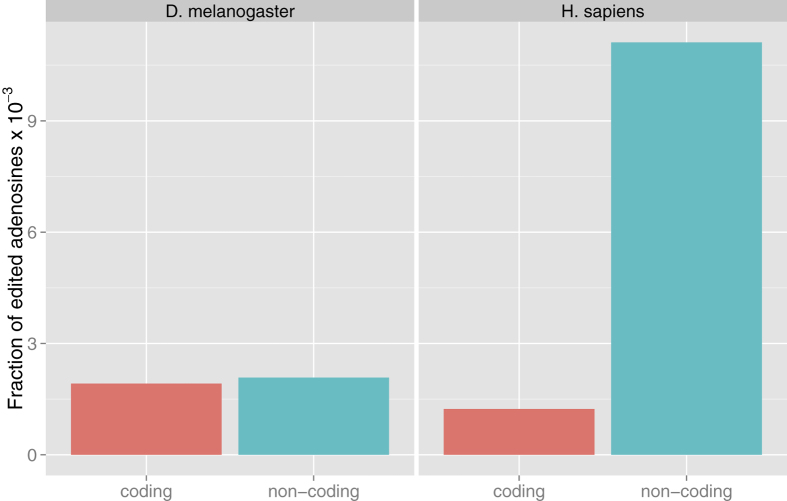
Distribution of editing events in coding and non-coding exonic regions in D. melanogaster and H. sapiens. In both cases, the frequency of events in coding and non-coding regions is statistically different, but the difference is larger in H. sapiens (p-value < 2.2*10^−16^) than in Drosophila (p-value = 0.01).

Both in the fly and in human, the frequency of edited adenines in non-coding regions is statistically different from that observed in coding regions (Fig. 1). Interestingly, the difference is more marked in H. sapiens (p-value < 2.2*10^−16^) than in Drosophila (p-value = 0.01).

### Residue specificity of editing events

**Figure 2:**
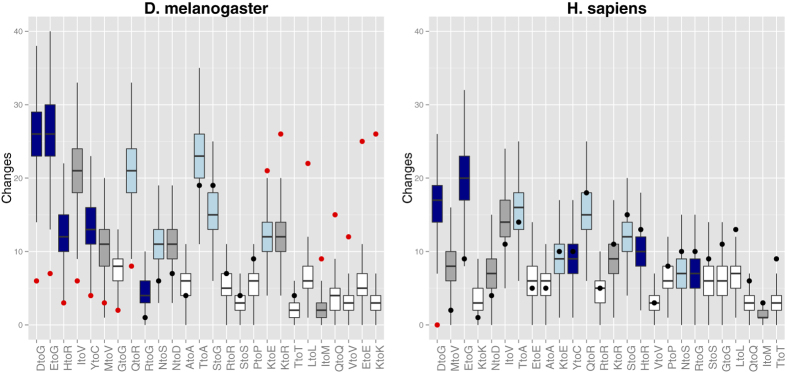
Residue specificity of editing events. The plots report the frequency of the observed editing events (dots) compared to what is expected by chance (boxplots) for D. melanogaster (**A**) and H. sapiens (**B**). Dark blue, light blue, dark grey and white boxes indicate very different, somewhat different, similar and synonymous amino acid changes, according to the residue change classification made by Laurent *et al.*^19^, respectively. In D. melanogaster synonymous changes are more frequent than expected, while nonsynonymous events leading to more drastic changes are less frequent. In H. sapiens the trend is similar, but the observed frequency occurrences are more similar to those expected by chance. Red dots highlight transitions with a frequency significantly different from random.

In order to verify whether editing in coding regions is a residue-specific phenomenon, we randomly generated reference datasets taking into account the codon composition of the proteins under examination. Each random dataset was built by randomly extracting codons containing at least one adenine from the coding regions present in our datasets and substituting the adenine with guanine. For both organisms we constructed one million such random datasets and subsequently compared the results with those experimentally observed. For each transition, we performed a Fisher’s exact test showing that some transitions have an occurrence significantly different from what expected by chance (Table 1 and Fig. 2).

As described in Methods, we excluded from our statistical analysis editing events mapping on alternatively spliced genes because, in these cases, one cannot know which transcript(s) is(are) affected by the editing event and this makes it unfeasible to generate the appropriate random model. Nevertheless we verified that the distribution of the various events observed in alternatively spliced transcripts (with the same reading frame) is not different from those observed in single transcripts (Tables S1 and S2).

Furthermore, we assessed the extent of deviation of the observed occurrences from the random ones by estimating a score (ER ratio) defined as the base-2 logarithm of the number of observed editing events divided by the number of random events times the total number of randomizations. A pseudo count of 1 was added to each value.

In D. melanogaster several events are observed with a frequency significantly different from that of H. sapiens for which only in one case the transition is significantly different from the expected value.

**Figure 3:**
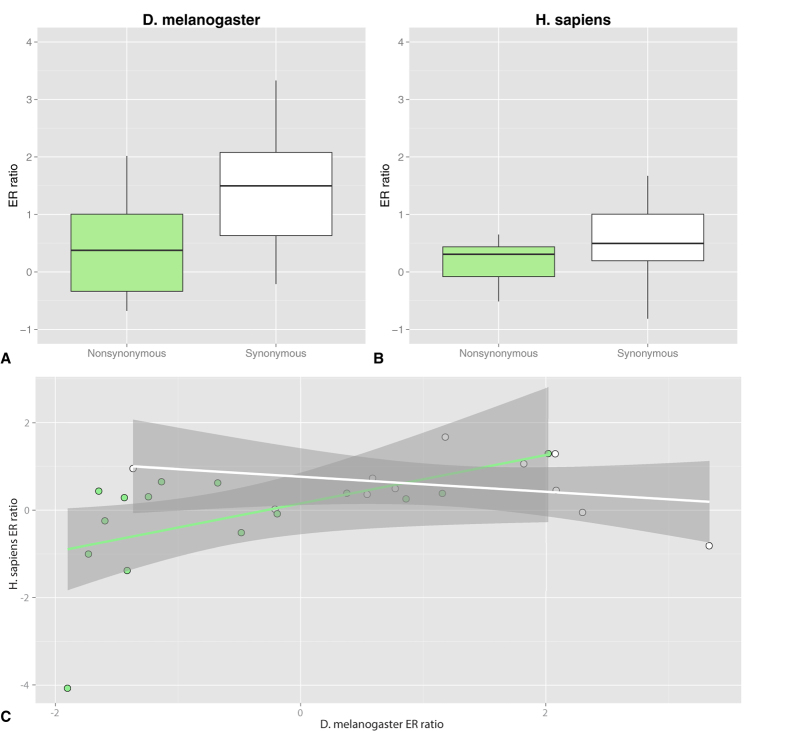
ER ratio of synonymous and nonsynonymous editing events in D. melanogaster and H. sapiens. Panels **A** and **B** report the ER ratio (see Methods) for synonymous and nonsynonymous events. In D. melanogaster the synonymous events have an ER ratio higher than nonsynonymous ones. (Wilcoxon-Mann-Whitney test p-value = 0.002). In H. sapiens the difference between nonsynonymous and synonymous events is not statistically significant. Panel **C** shows the correlation between the ER ratio of synonymous (white dots) and non synonymous (green dots) amino acid changes in human and fly (Pearson correlation test: rho = 0.52, p-value = 0.04). The synonymous events are not correlated. The shadowed areas indicate confidence intervals at 95%.

In D. melanogaster synonymous events tend to be favoured with respect to nonsynonymous ones (Fig. 3A, Wilcoxon-Mann-Whitney test p-value 0.002). On the contrary, in human the difference in ER ratio is not statistically significant (Fig. 3B, Wilcoxon-Mann-Whitney test p-value = 0.06).

The ER ratio of amino acid changes arising from nonsynonymous events in the two organisms are positively correlated (Pearson correlation test: rho = 0.52, p-value = 0.04) while this is not the case for synonymous events (Fig. 3C). This indicates that both organisms have the same tendency to avoid or favour the same nonsynonymous events. This may be due to the difference in the physical-chemical properties of the original and edited amino acids. Indeed, in both human and fly, we observe relatively more frequently editing events leading to similar amino acid changes while the less likely substitutions are those that produce a more drastic difference in the physical-chemical properties of the side-chains of the original and recoded amino acids (for example, D->G, see Fig. 2).

As mentioned above, the ER ratio of nonsynonymous substitutions in human and fly are correlated, however their absolute values are quite different (Wilcoxon-Mann-Whitney test W = 170, p-value 0.01, Figure S1) and lower in H. sapiens. Table 1 reports the ER ratio and p-values for all synonymous and nonsynonymous substitutions in human and fly.

### Properties of edited residues

We analysed the Uniprot^26^ database in order to verify whether residues subject to editing are enriched in specific functions in the protein (e.g. belong to active, binding or interaction sites or are targets of post-translational modifications, etc.) but we could not find any evidence for this hypothesis (data not shown). Furthermore, in both organisms, residues subject to editing do not have statistically significant different structural properties, such as solvent accessibility or tendency to be in specific secondary structure elements or in disordered/ordered regions, with respect to their non-edited counterparts (data not shown). We also tested whether editing is more frequently observed in genes coding for proteins belonging to specific functional classes in terms of their GO annotations^27^ in D. melanogaster, we found that they more frequently belong to four GO molecular function categories related to ion binding activity (Table S3) while we did not find any significantly enriched functional category for the human targets.

### Residue conservation

**Figure 4:**
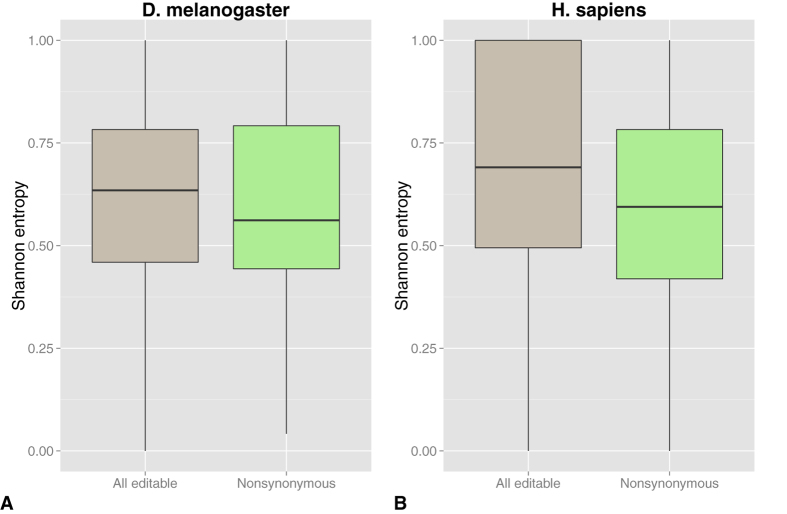
Conservation of edited residues. The Shannon entropy of the edited sites relative to nonsynonymous (light green) events is compared with that of all editable residues (gray). In D. melanogaster (**A**), sites affected by nonsynonymous editing have a level of conservation similar to that of all the editable ones. In H. sapiens (**B**), nonsynonymous editing affects residues with a level of conservation lower than other editable residues (Wilcoxon-Mann-Whitney test p-values = 2.3 × 10^−4^).

Conservation analysis is a useful tool for identifying functionally important residues in protein sequences^28^,^29^. We calculated the Shannon entropy for residues in multiple alignments of members of the family of the edited proteins^30^,^31^ as described in Methods. In D. melanogaster residues undergoing editing events that give raise to nonsynonymous changes have Shannon entropy similar to that of all the editable ones (Fig. 4A). The human dataset gives different results: the Shannon entropy of residues affected by nonsynonymous events is lower than that of all the editable ones (Wilcoxon-Mann-Whitney test p-value = 2.3*10^−4^, Fig. 4B).

One pivotal study^32^ identified 16 editing sites in the fly nervous system reporting that many of them occur at the level of conserved and functionally important amino acids. A comparative study on insect Kv2 K^+^ channels indicated that RNA editing usually occurs within highly conserved coding regions, but mostly alters less-conserved coding positions of these regions^33^. In the study of Laurent *et al.* nucleotides subject to nonsynonymous editing were found to be more conserved than other editable nucleotides randomly extracted from the analysed proteins. Our analysis of the fly dataset shows that this is not the case when the analysis of the conservation if performed at the residue level. Differently from the fly case, nonsynonymous editing events in H. sapiens are more frequently observed in less conserved regions of protein coding genes. These findings highlight a further difference between human and fly as far as editing in coding regions is concerned.

There is essentially no superposition between the edited targets in human and fly in terms of their orthology. We only found three pairs (data from Ensembl Compara ver77) of proteins edited in both species that are orthologous to each other. Their sequence alignment, however, showed that the corresponding edited residues are not aligned.

### Codon usage

Interestingly, all five synonymous editing events observed more frequently than randomly expected in D. melanogaster give raise to the substitution of a less frequently with a more frequently used codon. The opposite case, from a more frequently to a less frequently used codon, is instead observed much less frequently than randomly expected. This phenomenon is restricted to the fly and does not occur in human (Tables S4 and S5).

## Discussion

Several studies suggest that an important function of ADAR mediated RNA editing is to target noncoding dsRNAs, such as those made from repetitive elements of transposons and retrotransposons^34^,^35^,^36^ as well as pri-miRNAs^37^ possibly to counterbalance the efficacy of RNAi in the animal kingdom along with the expansion of repeat elements in the genome^38^. A recent work^39^ suggests a role of ADAR also in modulating inflammation and innate immunity. It has also been reported that RNA editing can give raise and/or eliminate splicing sites and a limited number of functional RNA editing events acting at the level of protein coding genes have been reported^40^,^41^,^42^.

In this study we compared the distribution and effect of the RNA editing events in two organisms for which this post-transcriptional modification is very well studied and documented.

Our analysis revealed several differences between H. sapiens and D. melanogaster in terms of the location of the editing events, the type and level of conservation of the encoded amino acids and the relationship between codon changes and the organism codon usage.

D. melanogaster and H. sapiens have different preference for the location of edited sites. Specifically, in human, edited adenosines are much more frequent in non-coding than in coding regions, with respect to what is observed in Drosophila. In both cases, however, edited events happen more frequently in non-coding than in coding regions, thus suggesting that modifying the encoded protein is not, in general, the main function of this mechanism.

Another difference that emerges from our study regards the properties of the target residue. There is a common trend in the type of nonsynonymous substitutions in avoiding drastic amino acid changes, but this effect is more evident in D. melanogaster.

We also observed that, in H. sapiens, the frequency of nonsynonymous edited residues is not significantly different from random. This latter conclusion is different from what is reported in a recent work by Xu *et al.*^43^. Indeed, we use a different model. We randomly extract codons and assess whether they can give raise to synonymous or nonsynonymous events, while Xu and co-workers consider the expected frequency for the total pool of adenines. The difference of the two approaches is limited to cases of codons containing more than one adenine. In these cases, it essentially never happens that both substitutions give raise to synonymous amino acids substitutions while, if both adenines do, the nonsynonimous substitution is counted twice in the Xu *et al.* model and only once in our case. Since we are interested in a codon-based analysis, we selected to extract codons to avoid over-estimating the expected frequency of non synonymous substitutions.

The most frequently observed synonymous substitutions in D. melanogaster go from a less frequently used codon to a more frequently used one, while changes from a more frequent to a less frequent codon are statistically significantly avoided in this organism. Once again, this is not mirrored by what happens in H. sapiens. This points to a possible role of editing in translation efficiency, at least in Drosophila.

In summary, our data indicate that editing events are differently distributed in H. sapiens and D. melanogaster and, in particular, the latter organism seems to be more lenient to the effect of editing events, while H. sapiens seems to more efficiently counter select possibly harmful events.

There can be several reasons for this different behaviour, however one intriguing possibility might be related to the extent of editing of a transcript in the two organisms. Indeed Xu *et al.*^43^ reported that the level of editing in H. sapiens has a median of about 30% (data based on six datasets, three of which included in DARNED), while Laurent *et al.*^19^ report a value of about 15% for D. melanogaster. One could speculate that the lower extent of editing in the fly transcripts might contribute to render this organism more tolerant towards amino acid substitutions due to the editing mechanism.

Undoubtedly, other aspects contribute to the phenomenon, for example the stability of the RNA molecule, its secondary structure or translation efficiency and these mechanisms certainly exert their effect also in coding regions. At present it is very difficult to establish their relative weight and more data of high quality and appropriate analyses will be required. Our data nevertheless demonstrate that the hypothesis that the encoded amino acid plays a role in the location of an edited site is supported by our statistical analysis of the Drosophila data, while a similar conclusion cannot be reliably drawn for the editing events so far recorded in H. sapiens.

## Methods

### Datasets

The compilation of A-to-I editing events of D. melanogaster and H. sapiens was obtained from the study of Laurent *et al.*^19^ and from the DARNED database^22^, respectively.

The D. melanogaster dataset consists in 3581 editing events, located in transcripts and intergenic regions. 1015 of these affect protein-coding exons. We removed those with ambiguous annotations (i.e. conflict, nonstop, non start, stop read-through) and those falling outside the protein coding genes annotated in Flybase release 5.50^44^. This reduced the dataset to 956 events. All protein-coding transcripts present in the Flybase release 5.50 with the same CDS were merged in CDS transcripts. The RNA CDS editing subset only includes events mapping on unique CDS transcripts and consists of 146 nonsynonymous substitutions and 130 synonymous substitutions, occurring in 197 proteins in total.

In H. sapiens the initial set of 309720 human A to I editing sites was filtered by removing 26070 sites overlapping with known SNPs, sites annotated in cancer tissues and sites for which the position indicated in DARNED did not correspond to an adenine in the genome (UCSC hg19).

The remaining sites of the human CDS RNA editing dataset were mapped to the gencodeV19 transcriptome^45^ and only sites that unambiguously mapped to the CDS of completely annotated protein-coding transcripts were retained.

The final human CDS RNA editing dataset contains 218 editing events in 127 transcripts, 143 of which give raise to nonsynonymous events.

We also recorded, mapped and counted cases of editing events affecting more than one alternatively spliced transcript with the same reading frame in both organisms.

Genomic positions of human and fly editing datasets were analysed by using the PICMI^46^ webserver and Annovar^47^ software in order to assess the effects of these transitions on all the overlapping transcripts of the reference transcriptomes.

### Random model

The dataset of codons present in the edited CDS of H. sapiens and D. melanogaster including at least one adenine was used to build the randomly expected distributions of editing events. We randomly extracted as many codons from the pool of codons in the CDS as there are in our dataset. This procedure was repeated one million times. Subsequently each putative A to I transition was used to calculate the corresponding recoded amino acid. The probability that the observed occurrences of nonsynonymous and synonymous transitions are due to chance alone was computed using the Fisher’s exact test. Furthermore we defined the ER score as:


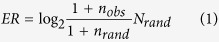


n_obs_ and n_rand_ are the number of observed editing events and the number of random events, respectively and N_rand_ is the total number of randomizations.

The perl script used to generate random datasets is available as additional information.

### Conservation of edited residues

The level of conservation of residues in the analysed datasets was estimated using the Shannon entropy^48^,^49^.

For each analysed transcript we retrieved members of their family from the HomoloGene database (build 67)^30^,^50^. Homologous protein sequences were aligned with Clustal Omega with default parameters^51^. The bio3d package^52^ was used to estimate the Shannon entropy (based on a 22 letter alphabet of 20 standard amino acids, plus a gap character and a mask character) for each aligned residue. Finally, the extent of conservation of nonsynonymous edited residues was compared to that of the editable residues present in our datasets (i.e. the codon of which contains at least one adenine that, if edited, leads to a nonsynonymous event). The statistical significance of the difference between edited and editable residues was assessed by the Wilcoxon-Mann-Whitney test.

## Additional Information

**How to cite this article**: Grassi, L. *et al.* RNA editing differently affects protein-coding genes in D. melanogaster and H. sapiens. *Sci. Rep.* **5**, 11550; doi: 10.1038/srep11550 (2015).

## Supplementary Material

Supplementary Information

Supplementary Information

## Figures and Tables

**Table 1:** ER Ratio and False discover rate for the amino acid substitution in D. melanogaster and H. sapiens.

	**ER ratio D. melanogaster**	**Corrected p-value D. melanogaster**	**ER ratio H. sapiens**	**Corrected p-value H. sapiens**	**type**
**KtoK**	3.33	2.57*10^−16^	−0.81	0.6	Synonymous
**EtoE**	2.3	3.21*10^−9^	−0.05	0.87	Synonymous
**VtoV**	2.08	2.88*10^−4^	0.46	0.87	Synonymous
**QtoQ**	2.07	3.86*10^−5^	1.29	0.26	Synonymous
**ItoM**	2.02	2.55*10^−3^	1.29	0.6	Similar
**LtoL**	1.82	8.34*10^−6^	1.06	0.14	Synonymous
**TtoT**	1.18	0.36	1.67	0.06	Synonymous
**KtoR**	1.15	8.93*10^−4^	0.38	0.73	Similar
**KtoE**	0.86	2.99*10^−2^	0.26	0.87	Somewhat different
**PtoP**	0.77	0.28	0.5	0.74	Synonymous
**StoS**	0.59	0.6	0.73	0.6	Synonymous
**RtoR**	0.54	0.55	0.36	0.87	Synonymous
**StoG**	0.38	0.42	0.39	0.7	Somewhat different
**TtoA**	−0.19	0.55	−0.08	0.87	Somewhat different
**AtoA**	−0.21	0.67	0.02	1	Synonymous
**NtoD**	−0.48	0.36	−0.51	0.67	Similar
**NtoS**	−0.68	0.19	0.62	0.6	Somewhat different
**RtoG**	−1.14	0.2	0.65	0.6	Very different
**QtoR**	−1.24	3.33*10^−3^	0.31	0.73	Somewhat different
**GtoG**	−1.37	4.52*10^−2^	0.95	0.26	Synonymous
**MtoV**	−1.41	2.12*10^−2^	−1.38	0.14	Similar
**YtoC**	−1.44	9.44*10^−3^	0.29	0.87	Very different
**ItoV**	−1.6	4.96*10^−4^	−0.24	0.73	Similar
**HtoR**	−1.65	4.75*10^−3^	0.44	0.73	Very different
**EtoG**	−1.73	3.86*10^−5^	-1.0	5.8*10^−2^	Very different
**DtoG**	−1.9	1.61*10^−5^	−4.07	1.5*10^−6^	Very different

The table reports the ER Ratio and Fisher’s exact test corrected p-value relative to each substitution in human and fly. Amino acid changes are classified as very different, somewhat different, similar and synonymous according to the residue change classification made by Laurent *et al.*^19^ Data are sorted according to the D. melanogaster ER ratio.
