# Supplementary material for: RNA editing differently affects protein-coding genes in D. melanogaster and H. sapiens
Source: Sci Rep. 2015 Jul 14;5:11550. doi: 10.1038/srep11550 (PMC4648400; doi:10.1038/srep11550)
Supplement: Supplementary Information [file srep11550-s1.pdf]

Supplementary material

RNA editing differently affects protein-coding genes in D. melanogaster and H. sapiens

Luigi Grassi,[1] Guido Leoni,[1] Anna Tramontano,[1,2]

[1]Deparment of Physics, Sapienza University of Rome, Piazzale Aldo Moro 5, 00185 Rome, Italy;
[2]Instituto Pasteur - Fondazione Cenci Bolognetti, Piazzale Aldo Moro 5, 00185 Rome, Italy;

List of Figures

1 ER ratio modules of nonsynonymous events 3

List of Tables

1 Editing events in D. melanogaster on all CDSs and on unique CDSs 4
2 Editing events in H. sapiens on all CDSs and on unique CDSs 5
3 Gene Ontology MF enriched terms 6
4 Synonymous codon frequencies in D. melanogaster 7
5 Synonymous codon frequencies in H. sapiens 8

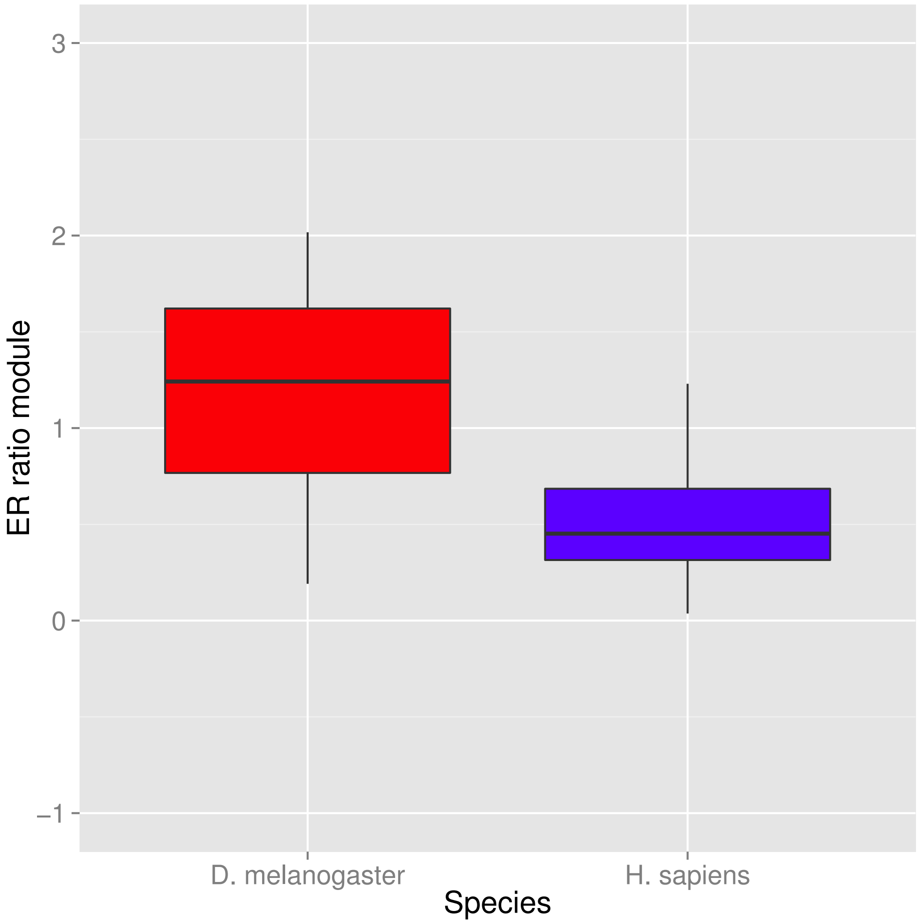

Supplementary Figure 1: **ER ratio modules of nonsynonymous events.** The ER ratio modules of nonsynonymous events in D. melanogaster and H.sapiens are shown as boxplots.

Event	Counts on all CDSs	Counts on unique CDSs
AtoA	21	4
DtoG	10	6
EtoE	63	25
EtoG	25	7
GtoG	10	2
HtoR	12	3
ItoM	37	9
ItoV	52	6
KtoE	37	21
KtoK	61	26
KtoR	92	26
LtoL	59	22
MtoV	15	3
NtoD	31	7
NtoS	46	6
PtoP	30	9
QtoQ	50	15
QtoR	73	8
RtoG	21	1
RtoR	16	7
StoG	77	19
StoS	15	4
TtoA	76	19
TtoT	13	4
VtoV	31	12
YtoC	36	4

Supplementary Table 1: **Editing events in D. melanogaster on all CDSs and on unique CDSs.** For each editing event (synonymous and nonsynonymous) the number of counts considering all the transcripts and only the ones with unique CDS are reported. The values are positively correlated (Pearson correlation test: rho=0.78, p-value = $3.2 * 10^{-6}$).

Event	Counts on all CDSs	Counts on unique CDSs
AtoA	26	5
DtoG	18	0
EtoE	28	5
EtoG	56	9
GtoG	23	11
HtoR	42	13
ItoM	12	3
ItoV	40	11
KtoE	61	10
KtoK	22	1
KtoR	74	11
LtoL	44	13
MtoV	13	2
NtoD	40	4
NtoS	35	10
PtoP	26	8
QtoQ	38	6
QtoR	83	18
RtoG	42	10
RtoR	23	5
StoG	96	15
StoS	50	9
TtoA	73	14
TtoT	32	9
VtoV	17	3
YtoC	31	10

Supplementary Table 2: **Editing events in H. sapiens on all CDSs and on unique CDSs.** For each editing event (synonymous and nonsynonymous) the number of counts considering all the transcripts and only the ones with unique CDS are reported. The values are positively correlated (Pearson correlation test: rho=0.78, p-value = $1.98 * 10^{-6}$).

GO CAT.	GO CAT. NAME	ADJ. PVALUE
GO:0043167	ion binding	1.49e-02
GO:0043169	cation binding	1.49e-02
GO:0046872	metal ion binding	1.49e-02
GO:0046914	transition metal ion binding	4.63e-02

Supplementary Table 3: **Gene Ontology MF enriched terms.**

Event	ER ratio	Corr. p-value	Codon change	Original codon freq.	Edited codon freq.	Ratio
AtoA	-0.21	0.67	GCAtoGCG	0.18	0.19	1.1
EtoE	2.3	$3.21 * 10^{-9}$	GAAtoGAG	0.34	0.66	1.9
GtoG	-1.37	$4.52 * 10^{-2}$	GGAtoGGG	0.29	0.073	0.25
KtoK	3.33	$2.57 * 10^{-16}$	AAAtoAAG	0.3	0.7	2.3
LtoL	1.82	$8.34 * 10^{-6}$	CTAtoCTG	0.093	0.42	4.5
LtoL	1.82	$8.34 * 10^{-6}$	TTAtoTTG	0.053	0.18	3.4
PtoP	0.77	0.28	CCAtoCCG	0.26	0.29	1.1
QtoQ	2.07	$3.86 * 10^{-5}$	CAAtoCAG	0.31	0.69	2.2
RtoR	0.54	0.55	AGAtoAGG	0.094	0.11	1.2
RtoR	0.54	0.55	CGAtoCGG	0.16	0.15	0.94
StoS	0.59	0.6	TCAtoTCG	0.099	0.2	2
TtoT	1.18	0.36	ACAtoACG	0.2	0.26	1.3
VtoV	2.08	$2.88 * 10^{-4}$	GTAtoGTG	0.11	0.46	4.2

Supplementary Table 4: **Synonymous codon frequencies in D. melanogaster.** Among D. melanogaster synonymous editing events those with significant corrected p-value and positive ER ratio are all transitions from less frequent to more frequent codons. The GtoG event is the only one significantly less frequent than expected by chance and, interestingly, regards a transition from a more frequent codon to a less frequent one.

Event	ER ratio	Corr. p-value	Codon change	Original codon freq.	Edited codon freq.	Ratio
AtoA	0.02	1	GCAtoGCG	0.24	0.1	0.42
EtoE	-0.05	0.87	GAAtoGAG	0.43	0.57	1.3
GtoG	0.95	0.26	GGAtoGGG	0.26	0.25	0.96
KtoK	-0.81	0.6	AAAtoAAG	0.44	0.56	1.3
LtoL	1.06	0.14	CTAtoCTG	0.073	0.39	5.3
LtoL	1.06	0.14	TTAtoTTG	0.079	0.13	1.6
PtoP	0.5	0.74	CCAtoCCG	0.28	0.11	0.39
QtoQ	1.29	0.26	CAAtoCAG	0.27	0.73	2.7
RtoR	0.36	0.87	AGAtoAGG	0.22	0.21	0.95
RtoR	0.36	0.87	CGAtoCGG	0.11	0.2	1.8
StoS	0.73	0.6	TCAtoTCG	0.16	0.052	0.32
TtoT	1.67	0.06	ACAtoACG	0.29	0.11	0.38
VtoV	0.46	0.87	GTAtoGTG	0.12	0.46	3.8

Supplementary Table 5: **Synonymous codon frequencies in H. sapiens.** In H. sapiens there are no synonymous editing events with significant corrected p-value.
